# Continuous intake of Trehalose induces white adipose tissue Browning and Enhances energy metabolism

**DOI:** 10.1186/s12986-019-0373-4

**Published:** 2019-07-16

**Authors:** Chikako Arai, Norie Arai, Shigeyuki Arai, Chiyo Yoshizane, Satomi Miyata, Akiko Mizote, Aki Suyama, Shin Endo, Toshio Ariyasu, Hitoshi Mitsuzumi, Shimpei Ushio

**Affiliations:** HAYASHIBARA CO. LTD, 675-1 Fujisaki, Naka-ku, Okayama, 702-8006 Japan

**Keywords:** Trehalose, Adipocyte size, Beige adipocyte, UCP1, Temperature

## Abstract

**Background:**

Trehalose is well known as a functional disaccharide with anti-metabolic activities such as suppression of adipocyte hypertrophy in mice and alleviation of impaired glucose tolerance in humans. Recently, a new type of adipocyte beige cells, involved in so-called white adipocyte tissue (WAT) browning, has received much attention as a target for adaptive thermogenesis. To clarify the relationship between adipocyte hypertrophy suppression and beige cells involved in thermogenesis, we examined the effect of trehalose on the changes in beige adipocytes in mice under normal dietary conditions.

**Methods:**

Mice fed a normal diet were administered water containing 0.3% (W/V) trehalose for 16 weeks, 0.3% (W/V) maltose, or water without saccharide (controls). Body temperature and non-fasting blood glucose levels were measured every 3 weeks. After 16 weeks of these treatments, mesenteric and inguinal adipose tissues were collected for measuring adipocyte size, counting the number of UCP1 positive cells by image analysis, and preparing mRNA to analyze beige adipocyte-related gene expression.

**Results:**

Mice administered a continuous intake of trehalose exhibited a thermogenic ability as represented by an increase in rectal temperature, which was maintained at a relatively high level from 3 to 9 weeks and was significantly higher at 15 weeks in comparison with that of the maltose group. In addition to the reduced hypertrophy of mesenteric and inguinal adipose tissues, the trehalose group showed a significant increase in the rates of beige adipocytes in each WAT in comparison with those of the maltose and the water groups. Interestingly, a negative correlation was found between the mean cell sizes of adipocytes and the rates of beige adipocytes in the WAT. Furthermore, real-time PCR showed that the expression of *Cidea* and *Ucp1* mRNAs, which are markers for beige adipocytes in the inguinal adipose tissue, increased in the trehalose group.

**Conclusions:**

Continuous administration of trehalose to mice fed a normal diet induced WAT browning accompanied by suppression of white adipocyte hypertrophy, elevated body temperature and decreased blood glucose levels, which resulted in enhancement of energy metabolism. Therefore, we propose trehalose as a new type of thermogenic dietary component to prevent obesity by promoting WAT browning.

## Background

The continuous worldwide increase in obesity is a serious health problem [1]. One promising approach to tackling the obesity problem is to understand the control mechanisms of adipocytes. Adipose tissue comprises white adipocyte tissue (WAT) and brown adipocyte tissue (BAT), both of which are related to energy homeostasis. When excessive energy is ingested, WAT stores energy in the form of triglycerides in unilocular white adipocytes, and it secretes many adipokines such as tumor necrosis factor-α (TNF-α), interleukin-6 (IL-6), and plasminogen activator inhibitor-1 (PAI-1), which induce insulin resistance [1, 2]. In contrast, multiple local BAT dissipates excess energy through thermogenesis, based on its high mitochondrial content and the expression of uncoupling protein 1 (UCP1) [3, 4]. Induction of thermogenesis in BAT is a promising method for the treatment of obesity [5, 6]. However, the amount of BAT in adults is quite small [3, 7]. Recently, brown-like adipocytes called beige adipocytes were found in WAT, and they expressed UCP1 to dissipate energy, similar to brown adipocytes [8–10]. The inducing phenomenon of beige adipocytes in WAT is called “browning”. Induction of the browning increases energy consumption and could help to improve metabolic disorders [5, 6, 9].

Trehalose, a non-reducing disaccharide consisting of two D-glucose residues, has been associated with various biological effects, such as inhibition of bone resorption [11], suppression of inflammatory responses [12, 13], alleviation of Huntington’s disease [14], and inhibition of atherosclerotic plaque formation [15]. Recently, we demonstrated that trehalose has anti-metabolic abilities such as lowering insulin secretion [16, 17]. When obese mice were fed a high fat diet (HFD) and administered drinking water containing 0.3% (W/V) trehalose from 8 to 15 weeks, adipocyte hypertrophy was suppressed, insulin resistance was alleviated, glucose tolerance was improved [18, 19], and hypertrophy of the Langerhans islands was prevented [20]. Since glucose, maltose, high-fructose corn syrup, and fructose did not have such effects [18, 20], these effects were thought to be specific for trehalose.

Based on the results of an oral glucose tolerance test administered to mice fed a HFD and given drinking water containing trehalose for a long period, we demonstrated that one of the mechanisms of adipocyte hypertrophy inhibition is lowered insulin secretion, because insulin increases the accumulation of fat [18, 19, 21]. However, the other mechanisms by which trehalose inhibits adipocyte hypertrophy are not yet known.

On the other hand, in human studies, subjects who ingested 10 g/day of trehalose for 12 weeks showed improvement in glucose tolerance under normal dietary conditions [22]. Although this has not been evaluated yet, trehalose is considered to be able to suppress adipocytes even under normal conditions. For anti-obesity, it is necessary to suppress the transition from healthy to obesity as well as to improve from the obese state. Therefore, regulation of adipocytes in normal conditions, especially the increase of beige adipocytes, is important for anti-obesity. However, the effect of trehalose in normal state on adipocytes has not been clarified yet. Therefore, the purpose of this study is to examine the effects of trehalose on beige and brown adipocytes under the normal feeding conditions. Besides the measurement of adipocyte size in both mesenteric and inguinal adipose tissues, we examine the rates of UCP1 positive cells to analyze the induction of beige adipocytes.

## Materials and methods

### Animals

Ten-week-old female C57BL/6 J mice were obtained from CLEA Japan, Inc. (Tokyo, Japan) and fed a standard diet (CE-2; CLEA Japan, Inc.) and water *ad libitum *for a week. The mice were kept in a temperature-controlled room with a 12-h light cycle. This study was approved by the Laboratory Animal Care Committee of the Hayashibara Co., Ltd. (Okayama, Japan), and all animal experiments were conducted in accordance with the Guidelines for Care and Use of Laboratory Animals of the Hayashibara Co., Ltd.

### Test substances

TREHA™ (Hayashibara Co. Ltd.) was used as the source of trehalose. It contains more than 98.0% trehalose dihydrate. SUNMALT™-S (Hayashibara Co. Ltd.) was used as the source of maltose, containing at least 92% maltose monohydrate.

### Study design

**Fig. 1:**
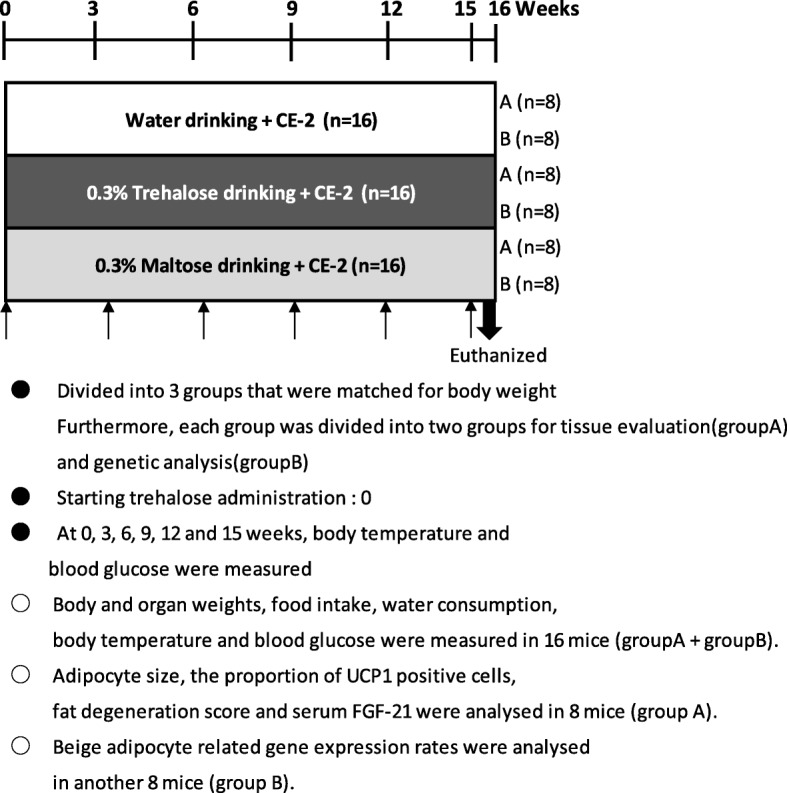
Experimental protocol. After a week of acclimation, all mice were divided into 3 groups matched by body weight and then administered 0.3% (W/V) trehalose or 0.3% (W/V) maltose in drinking water, or water alone. Interscapular and rectal temperature and non-fasting blood glucose were measured every 3 weeks (*n* = 16, group A + group B). The mice were euthanized at 16 weeks. Histological examination and analysis of adipose tissues and measurement of serum FGF-21 protein were performed using samples from 8 mice (group A). Analysis of gene expression in adipose tissues were performed another 8 mice (group B). FGF-21: fibroblast growth factor-21

The experimental protocol is shown in Fig. 1. A total of 48 mice were used in this study. After a week of acclimatization, the 11-week-old mice were randomly divided into 3 groups, which were matched for body weight. Detailed grouping is shown in Fig. 1. Two groups of mice were fed a commercial normal diet (CE-2) and then administered either 0.3% (W/V) trehalose or 0.3% (W/V) maltose in drinking water *ad libitum*, respectively (*n* = 16, for each group). As the experimental control, another group of mice received a normal diet and water alone (n = 16). Four mice were grouped per cage; each cage had to contain animals of a single group. Intake of food and water, which were replaced every other day, was monitored, and body weights were recorded weekly throughout the experiment. The skin temperature of the interscapular region was measured by an emission thermometer (#53006, Yokogawa Test & Measurement Corporation, Tokyo, Japan) every 3 weeks, the rectal temperature was measured using a rectal thermometer (KN-91-AD1687-M, Natsume Seisakusho Co., Ltd., Tokyo, Japan), and non-fasting blood glucose levels were measured with a simple blood glucose meter (Accu-Chek® Aviva Nano meter, Roche Diabetes Care Inc., Indianapolis, IN). After 16 weeks of treatment, the mice were euthanized under pentobarbital anesthesia. Adipose tissue was weighed, and blood samples were collected from the abdominal vena cava to measure fibroblast growth factor-21 (FGF-21). The FGF-21 was measured using an enzyme-linked immunosorbent assay kit (Mouse/Rat FGF-21 Immunoassay, R&D Systems Inc., Minneapolis, MN).

### Calculation of energy intake

The energy densities of the normal diet and of the drinking water containing 0.3% (W/V) trehalose or 0.3% (W/V) maltose were 14.2 kJ, 0.05 kJ, and 0.05 kJ per gram, respectively. Based on these data, the mean energy intake per mouse in each group was calculated using the following formulas:

Energy intake (kJ/mouse/day)

Mice consuming a normal diet and 0.3 % (W/V) trehalose or 0.3 % (W/V) maltose

Food intake (g) × 14.2 (kJ) + water intake (g) × 0.05 (kJ)

Mice consuming a normal diet and water

Food intake (g) × 14.2 (kJ) + water intake (g) × 0 (kJ)

### Histological analysis of adipocyte size

Mesenteric adipose tissue samples and inguinal adipose tissue samples were fixed with 10% (V/V) buffered formalin and embedded in paraffin. Sections were deparaffinized in xylene, stained with hematoxylin and eosin, and then examined using light microscopy. Photographs of the respective adipose tissue sections were taken of 5 random areas per section at 400 × magnification. More than 200 adipocyte sizes were measured by image analysis software (cellSens, Olympus Corporation, Tokyo, Japan).

### Rates of UCP1 positive cells in white adipose tissues

Sections of mesenteric adipose tissue and inguinal adipose tissue were stained with anti-UCP1 antibody (diluted at 1:200, ab23841, Abcam, Cambridge, UK) and then stained with secondary antibody (anti-rabbit diluted 1:2, Dako EnVision® + system-HRP labelled polymer; Agilent Technologies, Santa Clara, CA). Photographs were taken of 5 random areas of each adipose tissue sample at 400 × magnification. The number of UCP1 positive cells among the total adipocytes (500–800 cells) was measured by cellSens, and the rates of UCP1 positive cells were calculated.

### RNA extraction and quantitative real-time polymerase chain reaction (PCR)

Each mesenteric and inguinal adipose tissue was homogenized in the presence of QIAzol Lysis Reagent (Qiagen, Hilden, Germany) using a TissueRuptor® (Qiagen). The respective homogenates were separated into aqueous and organic phases by the addition of chloroform. According to the respective user manuals, total RNAs from the aqueous phase were isolated using an RNeasy Mini Kit (Qiagen) and DNase (Qiagen). Subsequently, first-strand cDNA was synthesized using SuperScript™ VILO™ Master mix (ThermoFisher Scientific, Waltham, MA). Specific primers for PCR analysis were designed using Primer 3 software (http://primer3.sourceforge.net/) (Table 1). The synthesized cDNA was mixed with SYBR™ Green Master Mix (ThermoFisher Scientific) and different sets of gene-specific primers. Real-time PCR was performed using a LightCycler® 480 system (Roche Diagnostic K.K., Tokyo, Japan). For quantitative purposes, the expression levels of the respective target genes were normalized to the housekeeping gene 18S rRNA.

**Table 1:** Summary of the sequences of gene-specific real-time PCR primers

Target	GenBank accession No.	Forward (5′ → 3′)	Reverse (5′ → 3′)
18S rRNA		ACTCAACACGGGAAACCTCACC	CCAGACAAATCGCTCCACCAAC
Cidea	NM_007702	ACAGAAATGGACACCGGGTA	TCCTTAACACGGCCTTGAA
Ucp1	NM_009463	GGGCCCTTGTAAACAACAAA	GAAGCCACAAACCCTTTGAA
Prdm16	NM_027504	CCAGATGTCAGCCATAGAAACC	TCTTGCCACAGTACCTGCAC
Pgc1α	NM_008904	ATGTGTCGCCTTCTTGCTCT	AGGGAGAATTGCGGTGTGT

### Statistics

The data is expressed as means ± standard deviations. A power analysis (G*Power 3.1.9.4, http://www.gpower.hhu.de/) showed that the sample size of 8 mice in each group was suitable for detecting a difference between the 3 experimental groups (1-β = 0.80, effect size = 0.7, α = 0.05). In addition, the calculated *p* values are described. Statistically significant effects of trehalose were examined using Tukey-Kramer (JMP 9.0: SAS, Cary, NC, USA) in the case of dispersion uniformity and normality. Non-parametric data were analyzed by the Steel-Dwass or Steel test or Wilcoxon test (JMP 9.0: SAS). A *p*-value less than 0.05 was considered significant.

## Results

### Continuous ingestion of trehalose showed no effect on energy intake, body weight, and tissue weight

**Fig. 2:**
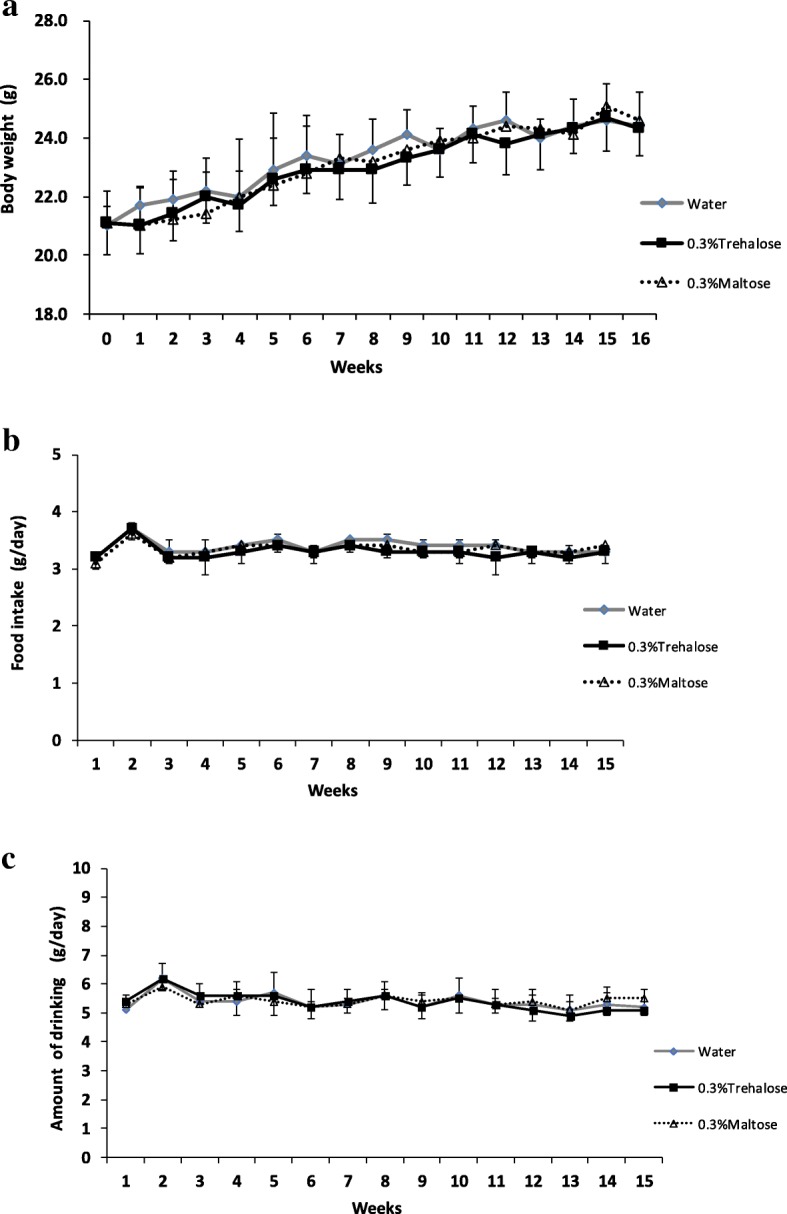
The effect of drinking water containing trehalose on body weight, food intake, and water consumption. The body weight (**a**), food intake (**b**) and water consumption (**c**) per mouse were measured every week during the experiment. Values were shown as means ± standard deviations (*n* = 16). Statistical analysis was performed using non-parametric Steel-Dwass. There was no significant difference among the three groups in the respective parameters (*p* < 0.05)

The energy intakes during the experiment were 48.1 ± 1.8, 47.2 ± 1.8, and 47.7 ± 1.6 kJ per mouse per day in the water, trehalose, and maltose groups, respectively. There was no significant difference in the energy intake between the trehalose and the maltose groups. Among the three groups, there were no significant differences in food and water consumption, and the weights of whole body and each organ (*p* < 0.05) are shown in Figs. 2a to c and Table 2.

**Table 2:** Body, adipose tissue and organ weights in mice after 16 weeks of trehalose intake

		Water	Trehalose	Maltose
Body weight (g)		24.6 ± 1.11	24.4 ± 1.20	24.3 ± 1.22
Adipose weight (g)	Mesenteric adipose	0.23 ± 0.05	0.23 ± 0.04	0.21 ± 0.05
	Perirenal adipose	0.15 ± 0.06	0.15 ± 0.05	0.14 ± 0.04
	Retroperitoneal adipose	0.34 ± 0.11	0.35 ± 0.12	0.31 ± 0.10
Liver (g)		1.28 ± 0.09	1.27 ± 0.15	1.24 ± 0.07

Values are means ± standard deviation of 8 mice per group. There are no significant differences between the 3 groups (*p* < 0.05). Statistical analysis was performed by non-parametric Steel-Dwass

### Trehalose exhibited thermogenic ability at core body sites

**Fig. 3:**
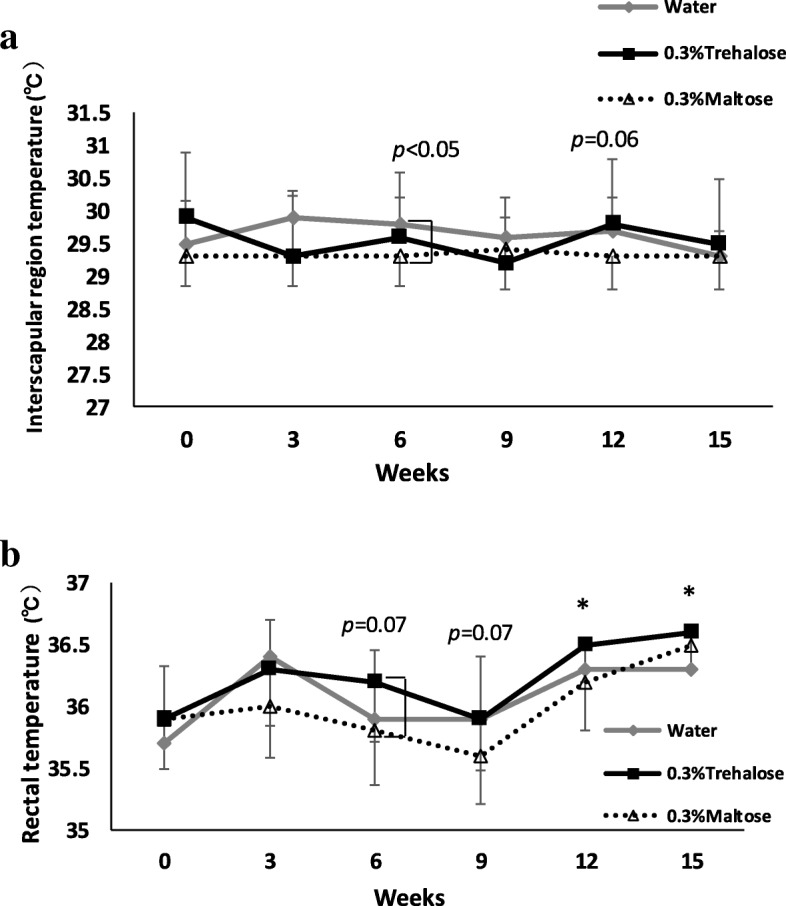
The effect of drinking water containing trehalose on body temperature. Every 3 weeks, interscapular (**a**) and rectal temperature (**b**) were measured. Values were shown as means ± standard deviations (n = 16). Statistical analysis was performed using Tukey-Kramer and Steel-Dwass. Interscapular temperature (**a**): Values show statistical significance at 6 weeks of the experiment (Water vs Maltose; *p* < 0.05). Rectal temperature (**b**): values show statistical significance at 12 weeks (Trehalose vs Maltose; *p* < 0.05) and at 15 weeks of the experiments (Trehalose vs Water; *p* < 0.05)

We first examined the thermogenic ability of trehalose by measuring body temperature in the scapular and the rectal regions. The surface temperature of the interscapular region showed no significant difference between the trehalose and the other two groups. The only observable difference was that the maltose group showed slightly lower temperatures at several points, as shown in Fig. 3a. When we measured the rectal temperature, trehalose appeared to exhibit thermogenic abilities, which were detected as a significantly higher temperature (*p* < 0.05) from 12 to 15 weeks in comparison with those of the maltose and the water groups, respectively. In addition, a tendency towards higher temperature (but not significant) was also observed in the trehalose group from 6 to 9 weeks (Fig. 3b).

### Blood glucose

**Fig. 4:**
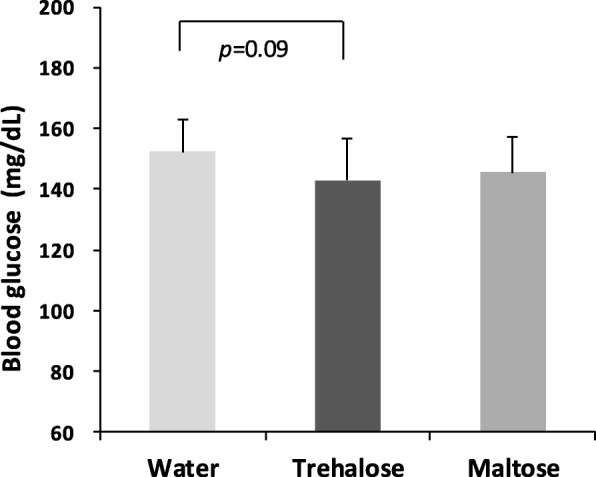
The effect of drinking water containing trehalose on blood glucose levels. Non-fasting blood glucose was measured after a light phase of 2 to 3.5 h at 15 weeks of the experiment. Values are shown as means ± standard deviations (n = 16). Statistical analysis was performed using Tukey-Kramer

When non-fasting blood glucose was measured after a light phase of 2 to 3.5 h at 15 weeks of the experiment, the blood glucose level of the trehalose group tended to be lower than that of the water group (*p* = 0.09), as shown in Fig. 4.

### Trehalose suppressed adipocyte hypertrophy in mesenteric and inguinal adipose tissues

**Fig. 5:**
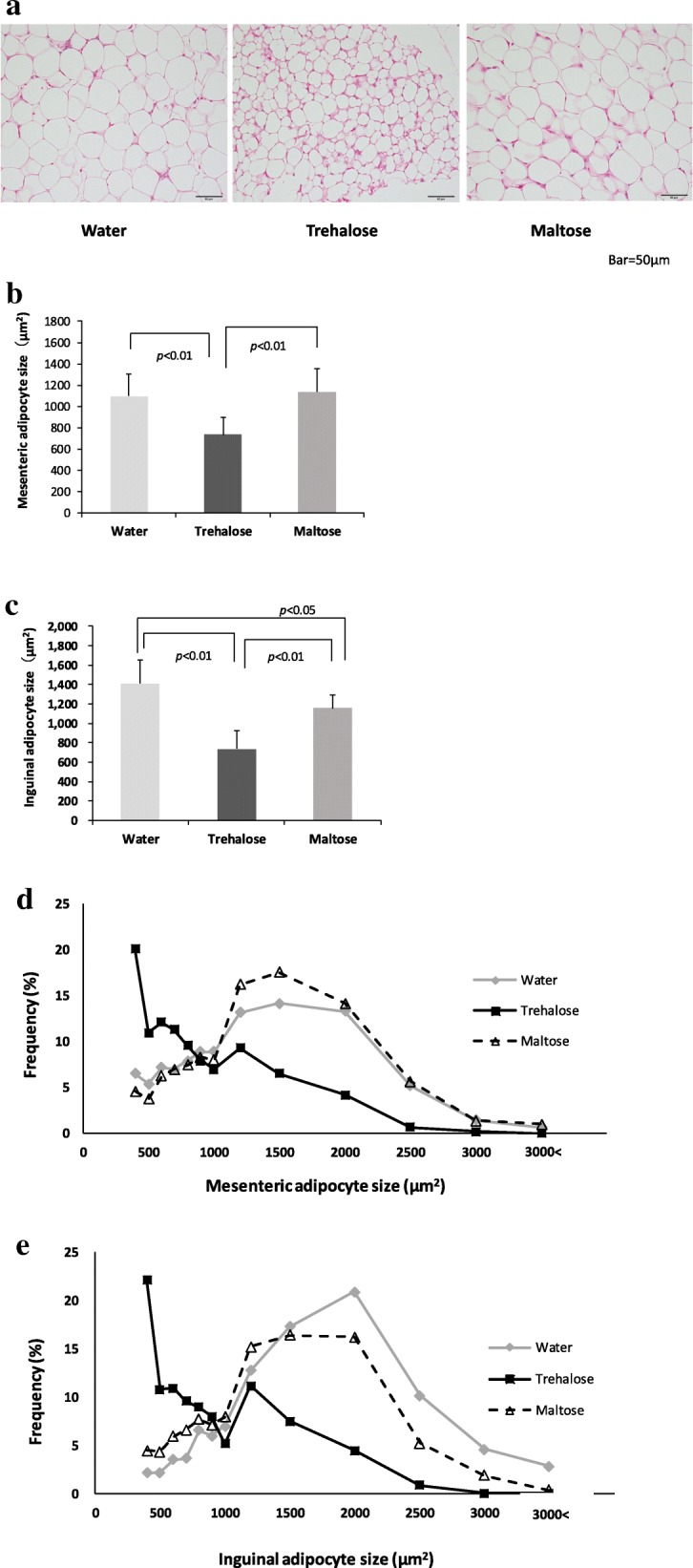
The effect of drinking water containing trehalose on adipocyte size in mesenteric and inguinal adipose tissue. The histology of WAT in the mesenteric and the inguinal adipose tissues and also the adipocyte size of both WATs were measured using the image software cellSens. Representative images of hematoxylin-eosin staining in sections of inguinal adipose tissue (× 400) are shown to assess histologically (**a**) and determine the size of mesenteric adipocytes (**b**) and inguinal adipocytes (**c**). In addition, cell size profiling in mesenteric adipose tissue (**d**) and inguinal adipose tissue (**e**) is summarized. Values are shown as means ± standard deviations (*n* = 8). Statistical analysis was performed with Tukey-Kramer. Values show statistical significance (5b and 5c; *p* < 0.01, 5c; *p* < 0.05). WAT: white adipose tissue

We examined the histology of the WAT in the mesenteric and the inguinal adipose tissues and measured the adipocyte sizes from both WAT using the image software cellSens. Histologically, the WAT in both the water and the maltose groups consisted of many large uniocular white adipocytes, whereas the WAT in the trehalose group consisted of many multilocular adipocytes with small lipid droplets (Fig. 5a). Surprisingly, even under the normal dietary conditions, the size of the mesenteric adipocytes in the trehalose group (732 ± 70 μm^2^) was significantly smaller than those of the water (1094 ± 212 μm^2^) and the maltose groups (1138 ± 218 μm^2^) (*p* < 0.01) (Fig. 5b). Moreover, the size of the subcutaneous inguinal adipocytes was also smaller in the trehalose (738 ± 184 μm^2^) group than in the water (1412 ± 244 μm^2^) and the maltose groups (1155 ± 136 μm^2^) (*p* < 0.01), as well as in the case of the mesenteric adipocytes (Fig. 5c). In both the mesenteric and inguinal adipose tissues, the peak of the frequencies of adipocyte size in the trehalose group was less than 400 μm^2^, whereas those of the water and maltose groups were from 1500 to 2000 μm^2^ (Fig. 5d and e).

### Trehalose increased the proportion of UCP1 positive cells in mesenteric and inguinal adipose tissues

**Fig. 6:**
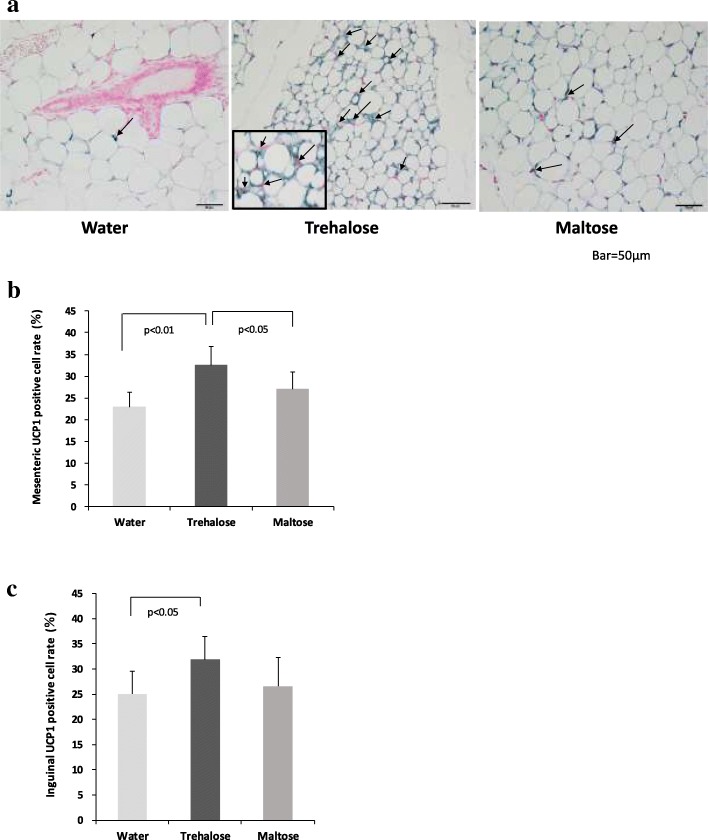
The effect of drinking water containing trehalose on browning in mesenteric and inguinal adipose tissue. UCP1 positive cells were stained blue with anti-UCP1 antibody (× 400) in the respective pictures (**a**). Arrows (→) indicate UCP1 positive cells. The inset is a magnified image of UCP1 positive cells (× 1000). The proportion of UCP1 positive cells in mesenteric adipose tissue (**b**). The proportion of UCP1 positive cells in inguinal adipose tissue (**c**). Values are shown as means ± standard deviations (n = 8). Statistical analysis was performed using Tukey-Kramer. Values show statistical significance (6b; *p* < 0.01, 6b and 6c; *p* < 0.05). UCP1: uncoupling protein 1

Next, we examined the numbers of beige cells contained in these adipose tissues. UCP1 staining was used to identify the beige cells in mesenteric and inguinal adipose tissues (Fig. 6a). As shown in Fig. 6a, UCP1 positive cells were stained blue in their cytoplasm. In the trehalose group, WAT was interspersed with a large number of multilocular cells, but those cells were scarce in WAT from the water and the maltose groups. The numbers of UCP1 positive cells were counted. The rates of the UCP1 positive cells in the total adipocytes are shown in Fig. 6b and c. In the mesenteric adipose tissue, a significantly higher rate of UCP1 positive cells was found in the trehalose group (32.6 ± 4.2%) than in the water (23.0 ± 3.4%) (*p* < 0.01) and the maltose groups (27.1 ± 3.9%) (*p* < 0.05) (Fig. 6b). Similarly, in the inguinal adipose tissue, the rate of UCP1 positive cells was significantly higher in the trehalose group (31.8 ± 4.6%) than in the water group (25.1 ± 4.5%) (*p* < 0.05), but that of the maltose group was not statistically different (Fig. 6c).

### Increased rates of UCP1 positive cells correlated with decreased adipocyte size

**Fig. 7:**
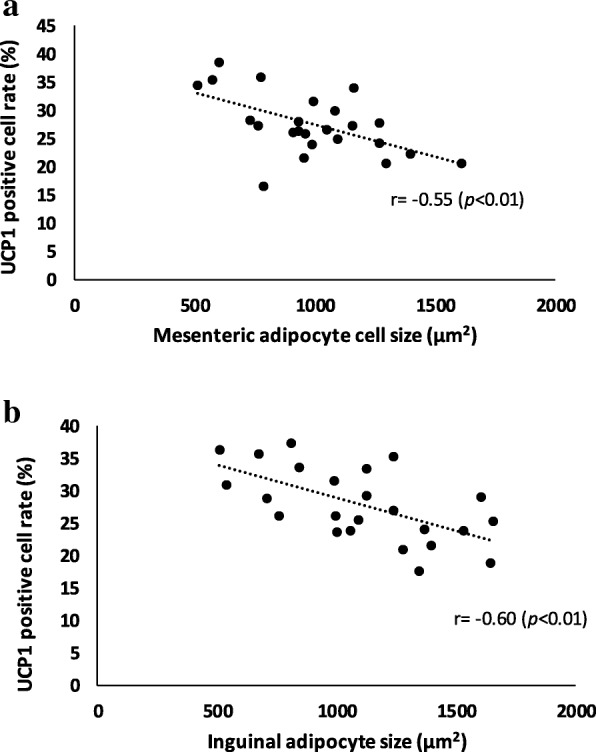
Correlation between UCP1 positive cell ratio (%) and white adipocyte size. The correlation between adipocyte size and UCP1 positive ratio (n = 8, each group) was determined in mesenteric (**a**) and inguinal (**b**) adipose tissue, respectively. Negative correlations were found between the adipocyte size and the proportion of UCP1 positive cells in both mesenteric and inguinal adipose tissue (Pearson correlation coefficients r = − 0.55, r = − 0.60, respectively). Values show statistical significance (7a and 7b; *p* < 0.01) (n = 8). UCP1: uncoupling protein 1

These results suggest that trehalose induced UCP1 positive beige cells in relation to the suppression of adipocyte hypertrophy. To confirm the relationship, therefore, we examined the correlation between adipocyte size and UCP1 positive ratio (*n* = 8, each group). In both the mesenteric and the inguinal adipose tissues, there was a negative correlation between the adipocyte size and the rates of UCP1 positive cells, and their correlation coefficients were r = − 0.55 (*p* < 0.01) and r = − 0.60 (*p* < 0.01), respectively (Fig. 7a and b). When more UCP1 positive cells were induced in the adipose tissues, it was found that the white adipocytes became smaller in size.

### Trehalose induced expression of beige adipocyte-related genes

**Fig. 8:**
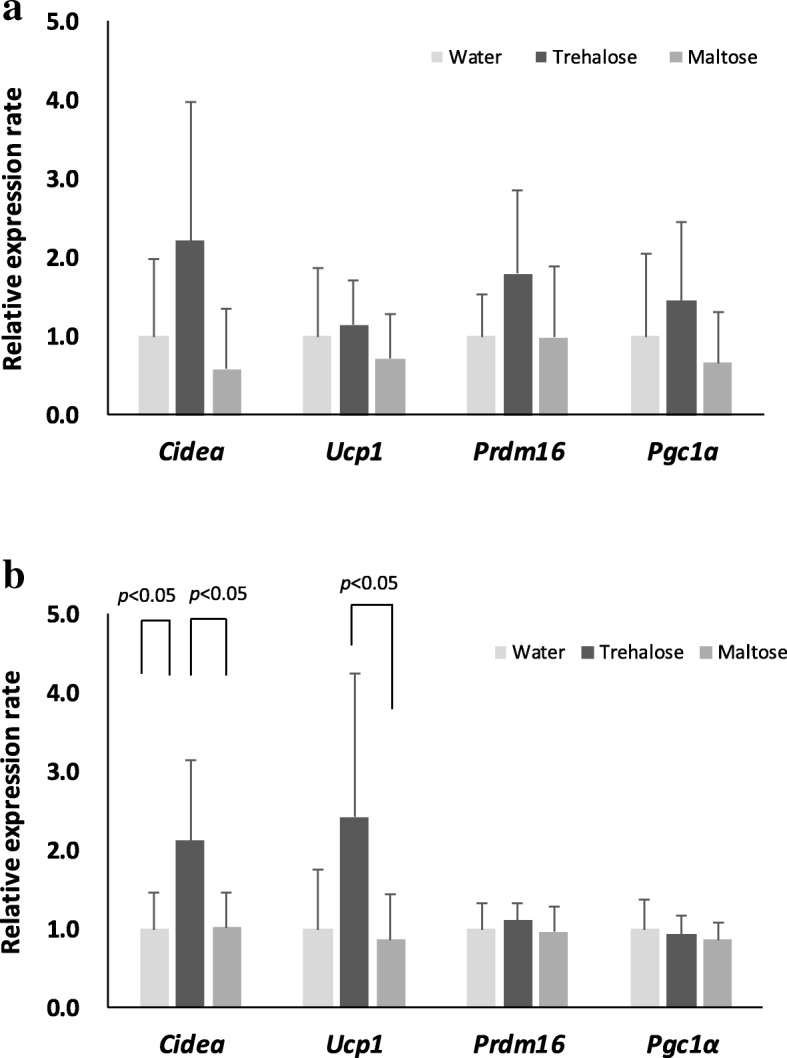
The effect of drinking water containing trehalose on beige-adipocyte-related gene expression. Total RNAs were extracted from mesenteric adipose tissues and inguinal adipose tissues, and the respective cDNAs were prepared as described in the manual. Subsequently, real-time PCR was performed using a LightCycler®480 system. For quantitative purposes, expression levels of the respective target genes were normalized to the housekeeping gene 18S rRNA. Mesenteric adipose tissues (**a**) and inguinal adipose tissues (**b**) are shown. Values are shown as means ± standard deviations (n = 8). *Cidea* expression was significantly higher in the trehalose group compared with both the water and the maltose groups (8b; *p* < 0.05, Steel). *Ucp1* expression was significantly higher in the trehalose group compared with the maltose group (8b; *p* < 0.05, Tukey-Kramer). *Cidea*: cell death-inducing DNA fragmentation factor-α-like effector A; UCP1: uncoupling protein 1

Furthermore, we measured the expression of genes associated with beige adipocytes. According to the previous paper [6, 23, 24], the four genes *Cidea* (cell death-inducing DNA fragmentation factor-α-like effector A)*, Ucp1, Prdm16* (PR domain containing 16)*,* and *Pgc1α* (PPARγ coactivator 1α) were selected as markers for beige adipocytes. *Cidea* is a gene that encodes a lipid droplet protein to regulate triglyceride deposition in adipocytes. *Prdm16* encodes a zinc finger protein and functions as a transcriptional coregulator to control the development of brown adipocytes. *Pgc1α* is a gene encoding a transcriptional coactivator and is defined as a master regulator of mitochondrial biogenesis. In the mesenteric adipose tissue, mRNA expression levels of *Cidea, Prdm16,* and *Pgc1α* in the trehalose group showed a tendency to be higher than those in the water and the maltose groups, although there were no significant differences (Fig. 8a). In the inguinal adipose tissue, the *cidea* expression level in the trehalose group was significantly higher compared to those of the water and the maltose groups (*p* < 0.05). Among the other genes, the *Ucp1* expression level in the trehalose group was significantly higher than that in the maltose group (*p* < 0.05), but not in the water group (Fig. 8b).

### Trehalose did not induce more severe fatty degeneration than maltose

**Fig. 9:**
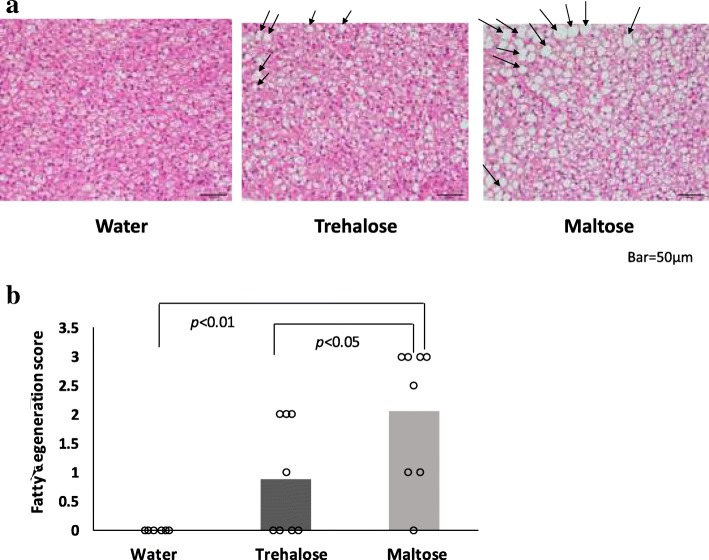
Effect of drinking water containing trehalose on BAT. Histological analysis of BAT (**a**) hematoxylin-eosin staining, × 400). Arrows (→) indicate vacuolation of BAT cells by fat deposition. Fatty degeneration scores (**b**) were graded as negative: 0, slightly: 1, mildly: 2, and severe: 3. Subsequently, the personal score was plotted (n = 8). Statistical analysis was performed using the nonparametric Wilcoxon test. Values show statistical significance (9b; *p* < 0.01 and *p* < 0.05). BAT: brown adipose tissue

We examined whether trehalose administration affected BAT, a representative heat-producing tissue. Since fatty degeneration of BAT was observed in the saccharide (trehalose or maltose)-administered groups, as shown in Fig. 9a, we compared the severity among the three groups by the histopathological method of fatty degeneration scores. In the water group, the BAT of all mice showed a normal structure and their fatty degeneration scores were zero. In contrast, both the trehalose and the maltose groups exhibited higher and varying degrees of fatty degeneration scores compared with those of the water group. However, the fatty degeneration score of the trehalose group was significantly lower than that of the maltose group (*p* < 0.05) (Fig. 9b).

### Trehalose had no effect on serum levels of FGF-21

**Fig. 10:**
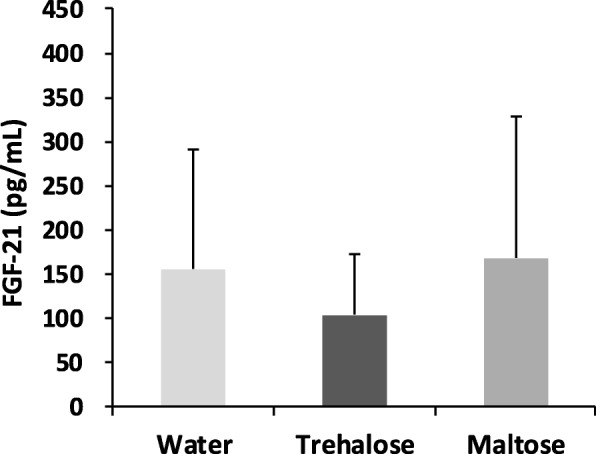
Effect of drinking water containing trehalose on FGF-21 protein levels in serum. Serum FGF-21 protein was measured at the end of the experiment. Values were shown as means ± standard deviations (n = 8). Statistical analysis was performed using non-parametric Steel-Dwass. FGF-21: fibroblast growth factor-21

FGF-21 is a circulating hepatokine that is a potential drug to regulate extrahepatic energy homeostasis and induce BAT activation or WAT browning. Therefore, serum FGF-21 levels were measured to investigate the possible involvement of FGF-21 in trehalose-induced browning. As shown in Fig. 10, the serum levels of FGF-21 were not different among the three groups.

## Discussion

In the present study, we demonstrated that drinking water containing trehalose with a normal diet significantly induced WAT browning in inguinal and mesenteric adipose tissues, with significant upregulation of the beige adipocyte-related genes *Cidea* and *Ucp1*. To our knowledge, this is the first report to evaluate the in vivo effects of disaccharide on WAT browning by immunohistochemical methods. We deduced that these effects resulted in elevated body temperature in the rectal region. Moreover, we found a strong correlation between the increase in the rate of UCP1 positive cells and the decrease in adipocyte hypertrophy, indicating that WAT browning is involved in the mechanism for the regulation of adipocyte size.

Regarding the UCP1 expression, there was a discrepancy between the gene and the protein expression levels in the mesenteric tissue, but this contradiction was not observed in the inguinal adipose tissue. The reasons are as follows: 1) samples from the mesenteric preparation may contain more non-adipose tissue than those from the inguinal preparation and 2) *Ucp1* gene expression is more inducible in the inguinal adipose tissue than in the mesenteric adipose tissue [25, 26].

It is well known that there are three possible ways to induce thermogenesis: 1) increase of BAT activity in existing classical brown adipocytes, 2) induction of formation of new adipocytes from adipogenic progenitor cells, and 3) switching of existing white adipocytes to beige adipocytes in subcutaneous fat (WAT browning). We provided evidence that trehalose exerts thermogenic activity through the WAT browning mechanism. It is unlikely that trehalose promoted BAT activity, as the histological analysis of BAT did not show such evidence in our experiments. Although the second possibility of progenitor cell differentiation is quite interesting, further investigation is required to verify the possibility.

In general, thermogenic stimulation such as cold exposure exerts its effects by stimulating the sympathetic nervous system (SNS) to release norepinephrine and then increasing the thermogenesis of BAT through β3-adrenergic receptor (adrb3) activation. Several dietary components have been described as thermogenic molecules exhibiting browning ability, including capsaicin, resveratrol, curcumin, green tea, berberine, and fish oil [27, 28]. Capsaicin and fish oil exert thermogenic effects by indirect activation of the adrb3, which is first induced by their binding to transient receptor potential vanilloid 1 and is followed by stimulation of SNS and release of norepinephrine. Curcumin and green tea have been shown to be effective in inducing WAT browning and thermogenesis through the mechanism of mitochondrial biogenesis and adrb3 activation. Since trehalose could directly induce browning in adipocytes in cell culture (data not shown), we deduced that the action of trehalose was not mediated by the SNS. It has also been suggested that resveratrol increases thermogenesis by stimulating mitochondrial biogenesis and increasing beige adipocytes. The underlying mechanism is thought to involve the activation of AMP-activated protein kinase (AMPK) and its downstream pathway to nicotinamide adenine dinucleotide (NAD)-sirtuin1-PCG1α [23, 29]. Furthermore, berberine, an alkaloid compound found in various herbs, has been shown to regulate adaptive thermogenesis, and that mechanism may involve AMPK and *Pgc1*α activation. Since we have previously demonstrated that trehalose increases serum high molecular weight adiponectin and mitigates insulin resistance in HFD-fed mice [19], on the other hand, the possibility to activate AMPK-*Pgc1α* downstream of adiponectin was also considered. As another mechanism, berberine has been reported to decrease inflammatory markers such as TNF-α and IL-6, and those anti-inflammatory effects are presumed to enhance WAT browning by M2 macrophage polarization [30]. Since trehalose could also exert anti-inflammatory effects, we predicted these effects in WAT browning. However, histological observations in our studies did not show significant infiltration of eosinophils and M2 macrophages in WAT.

Recently, Zang et al. [31] demonstrated that trehalose induces thermogenesis in a hepatocyte transcription factor EB (TFEB)-dependent manner, with a concomitant upregulation of *Ucp1* expression in hepatic and white adipose tissue. They proposed a mechanism in which trehalose first induces the hepatic fasting response by inhibiting the glucose transporter hepatocyte solute carrier 2A (SLC2A), and subsequently triggers AMPK-*Pgc1*α-TFEB-FGF-21 signaling. In this mechanism, hepatocytes are an important part of the action of trehalose, and systemic thermogenesis is supposed to be partially induced by the hepatocyte-centered fasting-like response. Therefore, we measured FGF-21 protein, which plays a central role in systemic thermogenesis. However, the serum FGF-21 protein was not elevated by trehalose administration in comparison to the water or the maltose groups. We do not know the exact reason for this discrepancy with the results of Zang et al. [31], but we assume that differences in the experimental conditions caused the inconsistency. In their experiments, 3% (W/V) trehalose was administered with drinking water for 5 days, while we administered 0.3% (W/V) trehalose for 16 weeks. That is, their administration was conducted with higher concentrations and for shorter periods of time compared with ours. Another possibility has been proposed by Fisher et al. [32]. They demonstrated that cold exposure induces browning in WAT without increasing the circulating FGF-21 levels, suggesting that upregulation of FGF-21 gene expression itself in WAT can operate thermogenesis signaling. However, it has not yet been possible to prove the involvement of FGF-21 in the mechanism of WAT browning by trehalose administration. Otherwise, our results may suggest the presence of distinct signal pathways.

On the other hand, Chevalier et al. [33] demonstrated that cold exposure leads to marked changes in the gut microbiota, and this “cold microbiota” phenomenon increases WAT browning. Therefore, we think that changes in the microbiota may be one of the browning mechanisms of trehalose. We are now planning some experiments to test our hypothesis.

## Conclusions

We have demonstrated that drinking water containing trehalose induced WAT browning accompanied with suppression of white adipocyte hypertrophy, elevation of body temperature and mitigation of blood glucose levels, even under normal diet conditions and at a lower concentration compared with previous studies. Therefore, we propose trehalose as a new type of thermogenic dietary component for improving human health by preventing obesity and promoting WAT browning.

## Data Availability

Not applicable.

## Abbreviations

adrb3: beta-3 adrenergic receptor
AMPK: AMP-activated protein kinase
BAT: Brown adipose tissue
*Cidea*: Cell death-inducing DNA fragmentation factor-α-like effector A
FGF-21: Fibroblast growth factor-21
HFD: High fat diet
IL-6: Interleukin-6
NAD: Nicotinamide adenine dinucleotide
PAI-1: Plasminogen activator inhibitor-1
PCR: Polymerase chain reaction
*Pgc1α*: PPARγ coactivator 1α
*Prdm16*: PR domain containing 16
SLC2A: Solute carrier 2A
SNS: Sympathetic nervous system
TFEB: Transcription factor EB
TNF-α: Tumor necrosis factor-α
UCP1: Uncoupling protein 1
V/V: Volume/volume
W/V: Weight/volume
WAT: White adipose tissue
